# Obesity-related subclinical hypothyroidism in childhood: Elevated triglycerides but not basal metabolic rate

**DOI:** 10.1038/s41390-024-03691-6

**Published:** 2024-11-05

**Authors:** Beata Tersander, Roger Olsson, Banu K. Aydin, Rasmus Stenlid, Iris Ciba, Hannes Manell

**Affiliations:** 1 Department of Women’s and Children’s Health, Uppsala University, Uppsala, Sweden; 2 Department of Medical Cell Biology, Uppsala University, Uppsala, Sweden

## Abstract

**Background:**

Studies on the associations between obesity-related subclinical hypothyroidism with basal metabolic rate and risk factors of cardiovascular disease in children and adolescents are scarce.

**Methods:**

Retrospective cohort study of children with obesity (*n* = 294) from the Uppsala Longitudinal Study of Childhood Obesity cohort. Differences in basal metabolic rate quantified by indirect calorimetry, and the cardiovascular risk factors; body mass index, blood lipids, fasting and 2 h oral glucose tolerance test glucose, glycated haemoglobin and insulin resistance, between subjects with and without subclinical hypothyroidism were investigated. The associations of baseline thyroid stimulating hormone (TSH) and ΔTSH with change in cardiovascular risk factors over time were assessed.

**Results:**

Subjects with subclinical hypothyroidism had elevated triacylglycerides but no alterations in basal metabolic rate or other measured cardiovascular risk factors. ΔTSH was positively associated with Δtriacylglycerides, Δtotal-cholesterol and ΔLDL-cholesterol, independently of age, sex, Δbody mass index and ΔT_4_. In the subclinical hypothyroidism group, 92% of individuals normalised their TSH 0.9–2.9 years later.

**Conclusions:**

Children with obesity and subclinical hypothyroidism did not have an altered basal metabolic rate but elevated triacylglycerides. During the follow-up period, TSH changed in parallel with several blood lipids. Elevated TSH often normalised without pharmaceutical intervention within 3 years.

**Impact:**

- The present study found that subclinical hypothyroidism in paediatric obesity is related to elevated triglycerides.
- The present study found that subclinical hypothyroidism is not associated to basal metabolic rate in paediatric obesity.
- TSH change over time correlated with the change in triglycerides and LDL and total cholesterol.
- Among subjects with subclinical hypothyroidism at baseline 92% normalised without pharmaceutical intervention within 3 years.
- This research adds to the knowledge of the longitudinal, natural course of elevated TSH in paediatric obesity which is expected to help to make informed decisions regarding follow-up and evaluation of this patient group.

## Introduction

The prevalence of obesity in childhood is increasing globally, in 2020 it was estimated that 8% of girls and 10% of boys aged 5–19 had obesity.^1^ Previous studies point to a relationship between thyroid function and overweight and obesity.^2^ Several studies have shown that children and adolescents with obesity have elevated levels of thyroid stimulating hormone (TSH), as well as the thyroid hormones, triiodothyronine (T_3_) and thyroxine (T_4_), in comparison to normal weight children and adolescents.^3–7^ While it is well known that overt hypothyroidism with elevated TSH leads to weight gain, several studies have concluded that the isolated abnormal TSH value in children with obesity is more likely a consequence, since weight loss results in the normalisation of TSH.^3,6,8^

One of the primary roles of thyroid hormones is to regulate energy balance. To do so, they are involved in numerous metabolic pathways regarding energy expenditure and storage.^9^ In the instance of excess thyroid hormones, basal metabolic rate will rise due to the regulation of catabolic and anabolic processes regarding fat, protein and carbohydrates.^10^ Furthermore, it has been hypothesised that the elevation of TSH and thyroid hormones found in children with obesity could be an adaptive response to the obesity itself, aiming to increase basal metabolic rate and thereby limit availability of further storage of energy as adipose tissue.^8^ Regarding this potential relationship between subclinical hypothyroidism and basal metabolic rate, studies conducted on paediatric populations are rare.

Moreover, studies have described correlations between subclinical hypothyroidism and cardiovascular risk factors such as elevated triacylglycerides (TAG), low-density lipoprotein (LDL) and total cholesterol.^11–15^ Thus, it seems that children with obesity and subclinical hypothyroidism have a higher risk of developing metabolic comorbidities. These studies are mostly cross-sectional and studies of changes in TSH and cardiovascular risk factors over time are rare in children and adolescents.

The aims of this study were to investigate whether (1) children and adolescents with obesity and subclinical hypothyroidism have a lower basal metabolic rate than those with normal TSH, (2) subclinical hypothyroidism in children and adolescents with obesity is associated with cardiovascular risk factors, elevated blood lipids, blood glucose, and/or insulin resistance (3) baseline TSH correlates with change in body mass index (BMI) and/or cardiovascular risk factors over time and (4) whether change in TSH over time correlates with changes in BMI and cardiovascular risk factors over time.

## Methods

### Study design and subjects

The current study was a retrospective cohort study based on subjects from the ongoing Uppsala Longitudinal Study of Childhood Obesity at the paediatric obesity clinic at Uppsala University Children’s Hospital.^16^ Children under the age of 18 with severe obesity or with overweight/obesity and comorbidities are eligible for referral to the paediatric obesity clinic, and thus also for recruitment to the Uppsala Longitudinal Study of Childhood Obesity cohort. Inclusion criteria for this present study was fasting blood samples provided at the first visit to the clinic and a BMI standard deviation score (BMI-SDS) > 1. Exclusion criteria were missing TSH at first visit to clinic, low TSH according to the Uppsala University Hospital Laboratory reference interval (<0.7, <0.6 and <0.5 mIU/L for subjects <6 years, 6–11 years and >11 years respectively), self-reported thyroid disease requiring treatment at or diagnosed within three months of the baseline visit. In addition, for subjects with elevated TSH, medical records were screened for diagnoses and ongoing treatment of thyroid disease. The study is part of the ongoing Uppsala Longitudinal Study of Childhood Obesity that has ethical approval from the Swedish Ethical Review Authority (registration number 2012/318). All participants and their guardians have given written informed consent and are informed of the possibility of withdrawing their consent at any time.

### Data collection

The details of data collection were as previously described.^16^ Briefly, fasting blood samples from a peripheral vein were drawn by a registered nurse. After collection, samples were immediately transported for analysis at the Uppsala University Hospital Laboratory. The following fasting blood samples were used in the present study: glucose (mmol/L), glycated haemoglobin (HbA1c, mmol/mol), cholesterol (mmol/L), LDL, (mmol/L), high-density lipoprotein (HDL, mmol/L), TAG (mmol/L), T_3_ (pmol/L), T_4_ (pmol/L) and TSH (mIU/L). An oral glucose tolerance test (OGTT) was performed after the fasting blood samples were drawn, as previously described^16^. Homoeostatic model assessment for insulin resistance (HOMA-IR) was calculated by fasting glucose * fasting insulin (µIU/mL)/22.5. Basal metabolic rate (kcal/24 h) was measured by indirect respiratory calorimetry (Jaeger CPX, Vyaire Medical, Mettawa, IL) in the morning in fasting conditions. For the analysis, basal metabolic rate was related to total body weight (kg) and to fat free mass (kg). Fat free mass was calculated by combining Caliper skin fold measurements with bioelectrical impedance analysis.^17^

Subclinical hypothyroidism was defined based on the Uppsala University Hospital Laboratory reference intervals as elevated TSH (cut-off 6.0, 4.8 and 4.3 mIU/L for subjects <6 years, 6–11 years and >11 years respectively) and thyroid hormones above the lower margin of the reference interval (T_3_ ≥ 3.7 and ≥3.9 pmol/L for subjects <6 years and ≥6 years respectively and T_4_ ≥ 12.3, ≥12.5 and ≥13.0 pmol/L for subjects <6 years, 6–11 years and >11 years respectively)^18^. We did not apply an upper limit for TSH values, the highest value among study subjects was 7.8 pmol/L. Data on the presence of thyroid peroxidase antibodies was not collected as part of the Uppsala Longitudinal Study of Childhood Obesity cohort and thus we were not able to assess this in the current study.

### Statistical analysis

Two cohorts were formed, a cross-sectional and a longitudinal. The cross-sectional cohort represents data from the first visit to the clinic. The longitudinal cohort represents subjects from the cross-sectional cohort who also had a follow-up visit to the clinic with a minimum of six months between visits. The cross-sectional cohort was divided into two groups, with and without subclinical hypothyroidism. To evaluate whether parameters followed the Gaussian distribution or not, an examination of histograms and quantile-quantile plots was performed. Unpaired Student’s T-test or Mann–Whitney U-test was used as appropriate to test differences in the cross-sectional cohort at baseline between the groups with and without subclinical hypothyroidism. For the comparison of sex distribution, chi-squared tests were used. Linear regression models were created with either baseline TSH or ΔTSH as the dependent variables and the change over time in the cardiovascular risk factors as independent variable, each risk factor in a separate model. Additional multivariate regression models were created by adjusting these models for age and sex, ΔBMI-SDS and ΔT_4_. Statistical analyses were done using GraphPad Prism version 9.4.1 (San Diego, California) and Stata/BE 18.0 for Mac (StataCorp LLC, College Station, TX). P-values of <0.05 were considered statistically significant.

## Results

### Cross-sectional data

In total 294 subjects from the Uppsala Longitudinal Study of Childhood Obesity cohort were included in the cross-sectional cohort of this study. Among these, *n* = 145 had basal metabolic rate data available from the baseline visit. The prevalence of subclinical hypothyroidism was 13% (*n* = 38) at baseline. There were no significant differences in sex, age, BMI-SDS, T_3_ or T_4_ levels between the groups (Table 1). TAG were elevated in the group with subclinical hypothyroidism (*p* = 0.01), while the remaining analyses displayed no significant differences between the groups (Table 2). In the cross-sectional cohort the following variables had missing data: OGTT 2h-glucose (*n* = 32), HOMA-IR (*n* = 16), T_3_ (*n* = 6), T_4_ (*n* = 4), LDL (*n* = 2), HDL (*n* = 2), TAG (*n* = 1) and HbA1c (*n* = 2). For both basal metabolic rate/kg total body weight and basal metabolic rate/kg fat-free mass, no significant differences between the groups were seen (Table 3). The children and adolescents with basal metabolic rate data available were similar in age, BMI-SDS, T_3_ and T_4_, levels as the whole cross-sectional cohort, but the sex distribution was slightly skewed towards more males, but not statistically significantly (*p* = 0.13= (Table 3).

**Table 1:** Baseline characteristics of the cross-sectional cohort of children and adolescents with obesity, divided by subclinical hypothyroidism.

	Subclinical hypothyroidism (*n* = 38)	Normal TSH (*n* = 256)	*p*
Sex *n* (%) males	21 (55%)	145 (57%)	0.87
Age (y)	13.3 ± 3.2	13.0 ± 3.2	0.60
BMI-SDS	3.2 ± 0.6	3.2 ± 0.6	0.70
T_3_ (pmol/L)	6.5 ± 0.9	6.4 ± 0.8	0.55
T_4_ (pmol/L)	15.1 ± 1.9	15.5 ± 2.5	0.30
TSH (mIU/L)	5.4 (4.8–7.8)	2.7 (0.8–4.4)	<0.01

Unless otherwise stated numbers are mean ± SD or median with range.

**Table 2:** Cardiovascular risk factors in children and adolescents with obesity, divided by subclinical hypothyroidism.

	Subclinical hypothyroidism (*n* = 38)	Normal TSH (*n* = 256)	*p*
Cholesterol (mmol/L)	4.4 ± 0.9	4.2 ± 0.8	0.08
LDL (mmol/L)	2.9 ± 0.7	2.7 ± 0.7	0.06
HDL (mmol/L)	1.0 (1.0–1.2)	1.0 (0.9–1.2)	0.42
LDL/HDL	2.6 (2.1–3.4)	2.5 (2.0–3.1)	0.34
TAG (mmol/L)	1.2 (1.1–1.7)	1.1 (0.8–1.5)	0.01
Fasting glucose (mmol/L)	5.7 (5.4–5.9)	5.7 (5.4–6.0)	0.55
2 h OGTT glucose (mmol/L)	7.8 (6.7–8.4)	7.5 (6.8–8.7)	0.83
HbA1c (mmol/L)	35 (32–37)	35 (33–37)	0.99
HOMA-IR	5.2 (4.1–9.7)	5.4 (3.4–8.4)	0.60

Unless otherwise stated numbers are mean ± SD or median with interquartile range.

**Table 3:** Basal Metabolic Rate in children and adolescents with obesity, divided by subclinical hypothyroidism.

	Subclinical hypothyroidism (*n* = 19)	Normal TSH (*n* = 126)	*p*
Basal Metabolic Rate (kcal/24 h)	1824 ± 387	1899 ± 456	0.50
Basal Metabolic Rate (kcal/24 h/kg body weight)	22.1 ± 4.3	22.7 ± 4.5	0.58
Basal Metabolic Rate (kcal/24 h/kg fat free mass)	36.7 ± 6.5	37.3 ± 8.2	0.79
Sex n (%) males	9 (47%)	82 (65%)	0.13
Age (y)	12.9 ± 3.4	12.9 ± 3.4	0.95
BMI-SDS	3.3 ± 0.6	3.2 ± 0.5	0.70
T_3_ (pmol/L)	6.6 ± 1.1	6.5 ± 0.7	0.63
T_4_ (pmol/L)	15.3 ± 1.8	15.6 ± 2.2	0.59

Unless otherwise stated numbers are mean ± SD.

### Longitudinal data

The longitudinal cohort (*n* = 100) had a mean age of 12.3 ± 3.0 years and BMI-SDS of 3.2 ± 0.5 at baseline. The mean follow-up time was 1.7 ± 0.8 years (range 0.5–5.2 years). Mean TSH at baseline was 3.0 ± 1.1 mIU/L, T_3_ of 6.5 ± 0.8 pmol/L and T_4_ of 15.5 ± 2.3 pmol/L. The cohort consisted of 54 boys and 46 girls, and 13 had subclinical hypothyroidism at baseline.

There was no significant association between TSH at baseline and ΔBMI-SDS (*β* = 0.03, *p* = 0.43) and this did not change when including age, sex and ΔT_4_ in the regression model (Table 4). There was a negative association between baseline TSH and ΔTAG (*β* = −0.10, *p* = 0.02) and ΔHbA1c (*β* = −0.61, *p* = 0.03) that remained statistically significant after the inclusion of age, sex, ΔBMI-SDS and ΔT_4_ in the models. There was no association between baseline TSH and the change in any of the other cardiovascular risk factors over time (Table 4). There were significant positive associations between ΔTSH and Δtotal-cholesterol (*β* = 0.11, *p* = 0.02), ΔLDL- cholesterol (*β* = 0.10, *p* = 0.02), ΔLDL/HDL (*β* = 0.15, *p* < 0.01) and ΔTAG (*β* = 0.13, *p* < 0.01). Regression coefficients were 80% of the unadjusted coefficient for ΔLDL- cholesterol and ΔLDL/HDL, 91% of the unadjusted coefficient for Δtotal-cholesterol and 92% of the unadjusted coefficient for ΔTAG when including age, sex, ΔBMI-SDS and ΔT_4_ in the regression models and all associations remained statistically significant (Table 5).

**Table 4:** Regression analyses between baseline TSH and change in listed cardiovascular risk markers as dependent variables over an average follow-up time of 1.7 ± 0.8 years in children and adolescents with obesity (*n* = 100).

	Model 1		Model 2		Model 3		Model 4	
	*B*	*p*	*B*	*p*	*B*	*p*	*B*	*p*
Δ BMI-SDS	0.03	0.43	0.03	0.42	0.03^a^	0.42^a^	0.04^a^	0.32^a^
Δ total-cholesterol (mmol/L)	0.00	0.95	0.02	0.76	0.02	0.74	0.03	0.58
Δ LDL (mmol/L)	0.01	0.80	0.03	0.53	0.03	0.57	0.04	0.42
Δ HDL (mmol/L)	0.01	0.60	0.01	0.75	0.01	0.53	0.01	0.53
Δ LDL/HDL	−0.05	0.36	−0.03	0.57	−0.04	0.45	−0.03	0.60
Δ Triglycerides (mmol/L)	**−0.10**	**0.02**	−0.08	0.05	**−0.08**	**0.04**	**−0.08**	**0.04**
Δ Fasting glucose (mmol/L)	0.02	0.50	0.02	0.59	0.02	0.61	0.01	0.72
Δ 2 h OGTT glucose (mmol/L)^b^	0.13	0.58	0.14	0.56	0.16	0.50	0.16	0.51
Δ HbA1c (mmol/L)	**−0.61**	**0.03**	−0.54	0.06	**−0.61**	**0.03**	**−0.66**	**0.02**
Δ HOMA-IR^c^	−0.13	0.87	−0.02	0.98	0.00	1.0	−0.03	0.96

Statistically significant associations in bold.

Model 1: Univariate model with baseline TSH as dependent and listed cardiovascular risk factor as independent variable.

Model 2: adjusted for age and sex.

Model 3: adjusted for age, sex and ΔBMI SDS.

Model 4: adjusted for age, sex, ΔBMI SDS and ΔT4.

^a^Regression model not including ΔBMI SDS as dependent variable.

^b^*n* = 58.

^c^*n* = 61.

**Table 5:** Regression analyses between ΔTSH and change in listed cardiovascular risk markers as dependent variables over an average follow-up time of 1.7 ± 0.8 years in children and adolescents with obesity (*n* = 100).

	Model 1		Model 2		Model 3		Model 4	
	B	p	B	p	B	p	B	p
Δ BMI-SDS	0.05	0.12	0.05	0.11	0.05^a^	0.11^a^	0.04^a^	0.19^a^
Δ total-cholesterol (mmol/L)	**0.11**	**0.02**	**0.11**	**0.02**	**0.11**	**0.02**	**0.10**	**0.03**
Δ LDL (mmol/L)	**0.10**	**0.02**	**0.10**	**0.02**	**0.09**	**0.02**	**0.08**	**0.04**
Δ HDL (mmol/L)	0.00	0.75	0.00	0.85	0.01	0.70	0.01	0.69
Δ LDL/HDL	**0.15**	**<0.01**	**0.15**	**<0.01**	**0.13**	**<0.01**	**0.12**	**<0.01**
Δ Triglycerides (mmol/L)	**0.13**	**<0.01**	**0.13**	**<0.01**	**0.12**	**<0.01**	**0.12**	**<0.01**
Δ Fasting glucose (mmol/L)	−0.02	0.53	−0.02	0.59	−0.02	0.55	−0.01	0.72
Δ 2 h OGTT glucose (mmol/L)^b^	0.19	0.31	0.18	0.33	0.15	0.42	0.16	0.41
Δ HbA1c (mmol/L)	0.34	0.17	0.31	0.21	0.23	0.36	0.29	0.24
Δ HOMA-IR^c^	−0.84	0.19	−0.90	0.16	−1.1	0.09	−1.0	0.11

Statistically significant associations in bold.

Model 1: Univariate model with ΔTSH as dependent and listed cardiovascular risk factor as independent variable.

Model 2: adjusted for age and sex.

Model 3: adjusted for age, sex, and ΔBMI SDS.

Model 4: adjusted for age, sex, ΔBMI SDS and ΔT4.

^a^Regression model not including ΔBMI SDS as dependent variable.

^b^*n* = 58.

^c^*n* = 61.

In the group with subclinical hypothyroidism, *n* = 12 (92%) had normalised their TSH at the follow-up visit, while only one (8%) still had elevated TSH at the follow-up visit. The range of follow-up for the subjects with subclinical hypothyroidism was 0.9–2.9 years. In the group with normal TSH at baseline, *n* = 80 (92%) remained normal while the remaining *n* = 7 (8%) had developed subclinical hypothyroidism at the follow-up visit. The total number of subjects with elevated TSH at the follow-up visit were 8 (8%), comparable to the proportion with elevated TSH at baseline (13%) (Table 1). None of the subjects started thyroxine substitution therapy within the follow-up period.

## Discussion

The most important findings of the present study were that children and adolescents with obesity and subclinical hypothyroidism have similar basal metabolic rate compared to those with normal TSH. Also, the longitudinal data indicate that a high TSH does not precede, but rather change in parallel with several cardiovascular risk factors independently of age, sex, change in BMI-SDS and change in T_4_. Lastly, most of the elevated TSH values normalised within a follow-up period of 0.9–2.9 years despite no pharmacological treatment.

One of the main aims of this study was to investigate the association between TSH and basal metabolic rate in childhood obesity. This to address the previously presented hypothesis that TSH rises in children with obesity with the aim to prevent further storage of energy in adipose tissue.^8^ The present study did not find a significant difference that could support this hypothesis, and this is in line with the results of one previous study in paediatric obesity.^11^ Another study did find a minor contribution to total energy expenditure by thyroid hormones in a group consisting of overweight and non-overweight children.^19^ Importantly, this is compatible with the results of the present study since TSH did not significantly correlate with thyroid hormones in our study population (data not shown). Taken together, it seems that the mild obesity associated TSH elevation is not closely related to variations in basal metabolic rate. This is also in line with adult data of basal metabolic rate and TSH in adults with obesity.^20^

TAG were significantly higher in the group with elevated TSH. The other blood lipids also tended to be higher in the group with elevated TSH, although not statistically significant. Beyond this, the regression analyses show that an elevation of TSH over time was associated with an elevation of TAG, cholesterol and LDL over time, independently of age, sex, ΔBMI-SDS and ΔT_4_. Higher levels of total-cholesterol,^11,12,21^, TAG^12^, and non-HDL cholesterol^21^ have previously been described in groups with elevated TSH. A large US study of children and adolescents also found an association between TSH and cardiovascular risk factors independently of BMI.^15^ Interestingly the increase in cardiovascular risk markers was seen within the upper normal range of TSH concentrations, further promoting the need for a reevaluation of reference values for thyroid samples.^15^ In summary, the results of the present study are in line with some previous research, showing that elevated TSH is linked to an unfavourable lipid profile, and therefore, likely also to a greater cardiovascular risk. How the lipid status is affected by overt hypothyroidism is well known, and arguably the same ought to happen, only to a lesser extent, in subclinical hypothyroidism.^22^ The result of the present study, and previous ones, may suggest this.^12,21^ TSH induces hepatic synthesis of cholesterol,^23^ while thyroid hormones increase clearance of LDL,^23,24^ decrease intestinal absorption of cholesterol,^25^ affect the metabolism of HDL^26^ and lower HDL.^27^ Thus, we consider it a reasonable interpretation that elevated TSH could potentially explain the lipid alterations seen in this and other similar studies. Furthermore, one might hypothesise that this lipid profile would normalise with levothyroxine substitution treatment.

Furthermore, a high TSH did not precede but rather change in parallel with blood lipids. The trend for BMI-SDS was similar, i.e., a change in parallel with TSH, but not statistically significant. Thus, it seems that in subjects with established obesity future changes in TSH are more closely correlated to change in lipid status than to the change in BMI. Taken together, our data points to it being more justifiable to treat elevated lipids and/or obesity than the subclinical hypothyroidism. The mechanisms behind the association between BMI and subclinical hypothyroidism remain elusive, although several hypotheses have been put forward, such as alterations in the hypothalamic-pituitary-thyroid-axis through leptin,^28^ variations in deiodinase activity,^29^ TH resistance at the pituitary level^30^ and impaired mitochondrial function.^30^

In >90% of the subjects with follow-up data available, the elevated TSH normalised between baseline and follow-up. However, the new percentage of elevated values remained roughly similar. One explanation for this could be a regression to the mean phenomenon with randomly caught elevated value as within-person variation in TSH known to exist in man.^31^ Thus, the result implies that for a child with obesity and slight TSH elevation it is quite reasonable to await repeated sampling prior to further intervention.

There are limitations to this study. The study had a retrospective study design, with the aim of this study being of an exploratory nature, another design of higher evidence would not be applicable. Another limitation is the relatively small group of subjects with subclinical hypothyroidism, especially in the group with basal metabolic rate data available, which would mean we have a low chance of detecting actual small differences and a limitation in drawing safe conclusions. However, our estimate was that the basal metabolic rate in those with subclinical hypothyroidism was ~97% of the basal metabolic rate in the non-subclinical hypothyroidism group, a difference that we would argue has little clinical significance. The screening tool for hypothyroidism present and under treatment at baseline in the Uppsala Longitudinal Study of Childhood Obesity cohort is a questionnaire, thus some failure to report might be present.

In conclusion, children and adolescents with obesity and subclinical hypothyroidism do not have an altered basal metabolic rate. However, they have a more unfavourable blood lipid profile. In addition, children and adolescents with obesity and elevated TSH levels do not gain more weight than those with normal TSH levels at baseline. Over time, TSH changes in parallel with the cardiovascular risk factors cholesterol, LDL and TAG independently of age, sex, ΔBMI-SDS and ΔT_4_. Furthermore, >90% of subjects with subclinical hypothyroidism at baseline normalises TSH spontaneously within three years.

## Data Availability

The datasets generated during and/or analysed during the current study are available from the corresponding author on reasonable request.
